# Investigation of glass forming ability of Al-based metallic glasses by measuring vaporization enthalpy

**DOI:** 10.1038/s41598-020-61134-8

**Published:** 2020-03-05

**Authors:** Byeong-uk Min, Jun-ho Lee, Ho-jun Park, Gyu-tae Jeon, Jae Im Jeong, Sung Hyuk Lee, Suk Jun Kim

**Affiliations:** School of Energy, Materials and Chemical Engineering, Korea University of Technology and Education, Cheonan, 31253 Korea

**Keywords:** Phase transitions and critical phenomena, Glasses, Characterization and analytical techniques

## Abstract

The analysis of the enthalpy changes for vaporization (Δ*H*_vap_) of Al-based metallic glass (MG) can provide insight into the origin of the MG’s glass forming ability (GFA). The Δ*H*_vap_ of three Al-based MGs, Al_84.5 ± *x*_(Y_10_Ni_5.5_)_15.5 ± *x*_, Al_85 ± *x*_(Y_8_Ni_5_Co_2_)_15 ± *x*_, and Al_86 ± *x*_(Y_4.5_Ni_6_Co_2_La_1.5_)_14 ± *x*_, (hereafter referred to as AYN_*x*_, AYNC_*x*_, and AYNCL_*x*_, respectively), is analyzed by measuring their weight losses below their glass transition temperatures. The relationship between Δ*H*_vap_ and aluminum concentration exhibit minimum values in the range of 83–85 at.% of Al, and the Δ*H*_vap_ increases, becoming saturated at 320–350 kJ/mol, as the percentage of Al deviates from this range. The depth of the enthalpy well, referring to the bottom of the parabolic graph of Δ*H*_vap_ against the Al concentration, is proportional to the viscosity of clusters showing liquid-like behavior. The amount of weight loss is proportional to the concentration of these clusters. The cluster viscosity and concentration influences the overall viscosity of the MGs, and thus determines the GFA.

## Introduction

Metallic glasses (MGs) are solid metallic materials, usually alloys, that have been cooled such that the material kinetically bypasses crystallization finishes in a disordered atomic-scale structure. They show certain properties of a liquid configuration, which can be confirmed by measuring viscosity in the temperature range from the glass transition temperature (*T*_g_) to the crystallization temperature (*T*_x_). This temperature range is referred to as the supercooled liquid region, and changes in viscosity that follow the Vogel-Tammann-Fulcher equation in this region are considered evidence of liquid-like behavior above *T*_g_^1^.

Below *T*_g_, MGs show both solid- and liquid-like behaviors. Relaxation, referring to the phase transition from glass to supercooled liquid or even to crystalline structure, is a particularly well-researched characteristic of MGs below *T*_g_^2–4^. The relaxation process releases heat, and the atomic motion during relaxation is similar to that in a liquid structure; the activation energy required for relaxation has been reported to be similar to that for diffusion in the glass phase^5^. Another liquid-like phenomenon observed from MGs is polymorphism, whereby MGs exhibit amorphous-to-amorphous phase transitions, as frequently observed in liquids and glasses, under extremely high pressures^6,7^. Certain crystallites such as iron and graphite also show polymorphism in crystallite-to-crystallite phase transitions that is also called allotropism. Therefore, the polymorphism of MGs can be considered evidence of both liquid- and solid-like behaviors. In other studies, MGs have exhibited both solid- and liquid-like behaviors simultaneously. In one study, 24.3% of the total volume of a Zr-based MG behaved like liquid and deformed anelastically whereas the remainder behaved as a solid^8^. Another study simulated an MG that consisted of two types of clusters, geometrically unfavored motifs and geometrically favored ones, and the former were found to provide better conditions for deformation, such that local regions with high concentrations of unfavored motifs behaved more like a liquid^9,10^. However, the percentage of the MG occupied by the unfavored motifs was not estimated in that study.

Here, we present thermodynamic evidence of liquid-like behavior from Al-based MGs below *T*_g_. We measured the enthalpy change for vaporization, Δ*H*_vap_, of the MGs. The MGs is the alloys with liquid structure and vaporization is universal phenomena in liquid. Enthalpy change for vaporization is the energy required for liquid-vapor phase transformation of the system. The system absorbs the energy at constant temperature and pressure, and the absorbed energy is used to increase the internal energy of the system and to expand the volume. On the atomic level, the energy is used to break the bonds. The bonding energy is related to interatomic or intermolecular force as a function of distance. These forces govern properties of materials such as vapor pressure, viscosity, surface tension, and capillary force. Especially, inverse proportional relationship between viscosity and vapor pressure from various liquids and liquid solutions was previously reported^11,12^. The enthalpy change of vaporization of MG should be an appropriate value to evaluate the properties of MGs with liquid structure. In this study, we measured ΔH_vap_ of the Al-based MGs: Al_84.5 ± *x*_(Y_10_Ni_5.5_)_15.5 ± *x*_; Al_85 ± *x*_(Y_8_Ni_5_Co_2_)_15 ± *x*_; and Al_86 ± *x*_(Y_4.5_Ni_6_Co_2_La_1.5_)_14 ± *x*_. These materials, hereafter referred to as AYN_*x*_, AYNC_*x*_, and AYNCL_*x*_, respectively, are shown to exhibit minimum Δ*H*_vap_ values when the Al concentration is in the range of 83–85 at.%, and because the Δ*H*_vap_ increases when the Al concentration deviates from that range, we call the parabolic curve an *enthalpy well*. We analyzed Δ*H*_vap_ by using thermogravimetric analysis and utilized the Langmuir equation that used to calculate the vapor pressure of tungsten^13^. The method described herein can be easily applied to evaluate MGs’ intrinsic properties and to optimize composition and fabrication processes for various applications.

## Results

**Figure 1:**
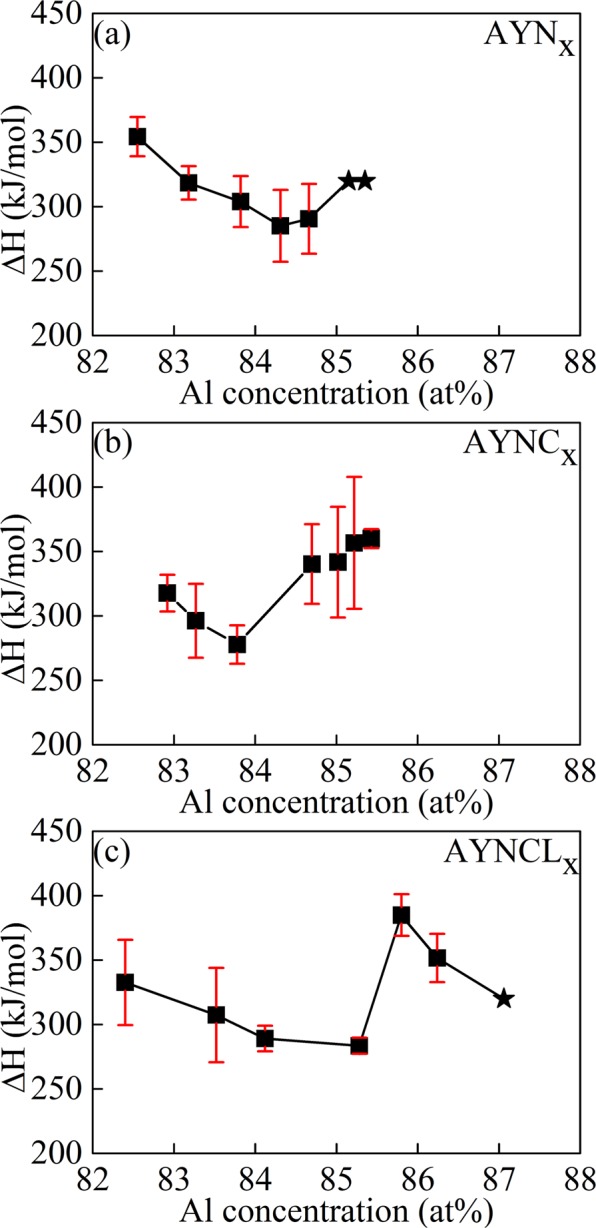
Δ*H*_vap_ vs. Al concentration of AYN_x_, AYNC_x_, and AYNCL_x_. The values of Δ*H*_vap_ at the Al concentrations of 85.2% and 85.4% (indicated by the stars in the Fig. 1) were hypothesized to be the same as enthalpy change for sublimation (Δ*H*_sub_) of crystalline Al because no weight loss was not detected from these samples.

The method of determining enthalpy changes is briefly introduced here and detailed below and in the Supplementary Information. The Δ*H*_vap_ was determined from

1 $$\mathrm{ln}({P}_{vap})=-\frac{\Delta H}{RT}+C$$

and obtained from the slope of ln(*P*_vap_) vs. 1/*T* graph. where *P*_*vap*_, *R*, *T*, and C are the vapor pressure, gas constant, temperature and constant, respectively. The vapor pressures of the MG ribbons were calculated by thermogravimetric analysis, in which the weight loss during isothermal annealing was measured at various temperatures below the crystallization temperature (*T*_x_). We used the following to calculate the Langmuir equation:

2 $$\frac{dm}{dt}=\alpha Z{P}_{vap}\sqrt{\frac{{M}_{A}}{2\pi RT}},$$

where *dm/dt*, *α*, *Z*, and *M*_*A*_ are the evaporation rate, vaporization constant, evaporation surface area, and molecular weight of the evaporating substance, respectively. α is the ratio of the measured vapor pressure to the equilibrium vapor pressure^14^. It is dependent on the environment of measurement^15^. In this study, this value was used to adjust the experimentally measured values to theoretical values. Further explanation for α was provided in Method section. In this study, molar mass of aluminum was used for *M*_*A*_ yet average molar mass of MG in *M*_*A*_ led to the same value of Δ*H*_vap_. Figure 1 shows the curves of Δ*H*_vap_ vs. Al concentration, which illustrate the enthalpy wells.

The widths and depths of the enthalpy wells of AYN_*x*_, AYNC_*x*_, and AYNCL_*x*_ depended on the number of solute elements, Y, Ni, Co, and La. As shown in Fig. 1, the Δ*H*_vap_ values at the bottoms of the parabolic curves were comparable to each other within the error range: for all three compositions, the Δ*H*_vap_ values at these points ranged from 275 kJ/mol to 285 kJ/mol. The minimum points of the enthalpy wells of AYN_*x*_ and AYNC_*x*_ were at approximately 84.0%, whereas the minimum of AYNCL_*x*_ was higher, at 85.3% of Al concentration. The Δ*H*_vap_ increased to saturation levels of 320–350 kJ/mol as the Al concentration deviated from that at which the enthalpy well occurred. The widths of the enthalpy wells of AYN_*x*_ and AYNC_*x*_ were comparable to each other, at approximately 2.0% (83–85 at.%), whereas that of AYNCL_*x*_ was wider, at approximately 2.8% (83–85.8%), and was extended in the direction of higher Al concentrations.

**Figure 2:**
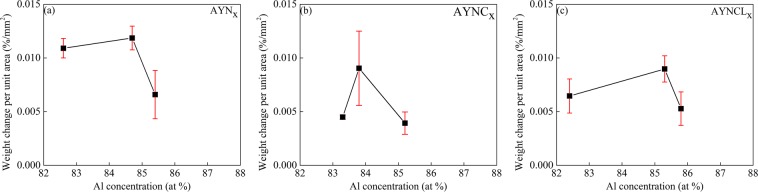
Weight loss of the MGs per sample area measured under continuous heating. Samples with compositions at the left edge, bottom, and right edge of the enthalpy wells of (**a**) AYN_*x*_, (**b**) AYNC_*x*_, and (**c**) AYNCL_*x*_ were selected for analysis. The measured TGA data is provided in Supplementary Fig. S6.

The weight losses of the Al-based MG ribbons were also analyzed under continuous heating mode to measure the total weight loss up to crystallization. The weight losses per unit area were measured from MG samples with compositions at the bottom and both edges of the enthalpy wells of AYN_*x*_, AYNC_*x*_, and AYNCL_*x*_, and Fig. 2 and Supplementary Fig. S1 show the measurement results. The sample weights decreased as temperature increased because of vaporization (Supplementary Fig. S1). The rate of decrease reduced near *T*_x_ and then became zero near 723 K. Above 723 K, the weights of all samples increased because of oxidation following crystallization^16,17^. The maximum weight loss per unit area was observed from the compositions at the bottoms of the enthalpy wells of AYN_*x*_, AYNC_*x*_, and AYNCL_*x*_. The weight loss up to 723 K of the compositions at the bottom of the enthalpy wells of AYN_*x*_, AYNC_*x*_ and AYNCL_*x*_ ranged from 0.008 to 0.011 wt.%/mm^2^. The samples with compositions at either edge of the enthalpy wells exhibited less total weight loss compared to the composition at the bottom for all three compositions. The total weight loss vs Al concentration data was inverse proportional to the enthalpy well curves.

**Figure 3:**
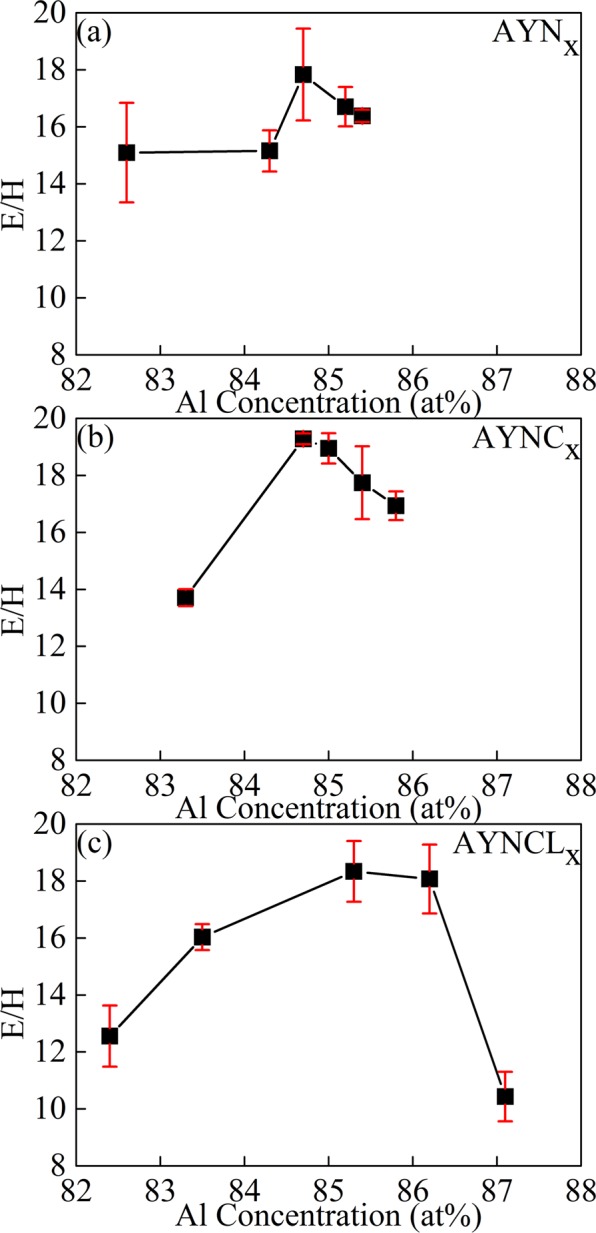
Ratios of elastic modulus to hardness (E/H) of (**a**) AYN_*x*_, (**b**) AYNC_*x*_, and (**c**) AYNCL_*x*_, measured using a nanoindenter.

The relative glass-forming abilities (GFAs) of AYN_*x*_, AYNC_*x*_, and AYNCL_*x*_ were analyzed to investigate the relationship between the GFA and the enthalpy wells. Al_84.5_Y_10_N_5.5_, Al_85_Y_8_N_5_Co_2_, and Al_86_Ni_6_Y_4.5_Co_2_La_1.5_ have been reported to represent the maximum GFAs in AYN_*x*_, AYNC_*x*_, and AYNCL_*x*_, respectively; their GFAs are 0.75, 0.90, and 1.00 mm, respectively^18–20^. In this study, the compositions with the highest GFAs were confirmed by measuring the ratios of elastic modulus to hardness (E/H) using a nano-indenter. The maximum E/H values of typical MGs with representative compositions are in the vicinity of 20^21^. The results of this study, obtained from Al_84.7_(Y_10_Ni_5.5_)_15.3_, Al_84.7_(Y_8_Ni_5_Co_2_)_15.3_, and Al_85.3_(Y_4.5_Ni_6_Co_2_La_1.5_)_15.7_ (AYN, AYNC, and AYNCL, respectively, and their compositions were similar to the Al_84.5_Y_10_N_5.5_, Al_85_Y_8_N_5_Co_2_, and Al_86_Ni_6_Y_4.5_Co_2_La_1.5_) showed maximum E/H values. The respective maximum E/H values of MGs were 17.8 ± 1.61, 19.3 ± 0.2, and 18.6 ± 0.3. These values decreased as the Al concentration deviated on either side, as shown in Fig. 3. Thus, the E/H values were almost inversely proportional to the enthalpy wells. In addition, the E/H measurements matched the reliability of the relative GFAs and compositions.

The Δ*H*_vap_ of AYN was lower than those of AYNC and AYNCL because of their positions on the enthalpy wells. Whereas AYN, the composition with the maximum GFA of AYN_x_, was found within the enthalpy well, AYNC and AYNCL were found near the right edge of their enthalpy wells. Because of the difference in their positions in relation to the enthalpy wells, the Δ*H*_vap_ of AYN was 290 ± 27 kJ/mol, which was lower than those of AYNC (340 ± 43 kJ/mol) and AYNCL (384 ± 16 kJ/mol.) Therefore, it can be thought that this slight mismatch between the Al concentrations of the composition at the bottom of the enthalpy well and those with the maximum GFA contributes to the higher GFAs of AYNC and AYNCL compared to AYN.

## Discussion

To understand the relationship between the enthalpy wells and GFA, the atomic structure of the MGs should first be considered. Ma *et al*. previously proposed that MGs consist of clusters of geometrically unfavored motifs (GUMs) or geometrically favored motifs (GFMs)^10,22,23^. The clusters are composed of individual solute atoms (Ni, Co, Y, and La) surrounded by a number of Al atoms. The number of Al atoms is defined as a coordination number, and this parameter exhibits a Gaussian distribution. Each solute atom has a preferred coordination number for glass formation, the average values of which are 9.4, 9.4, 16.9, and 17.5 for Ni, Co, Y, and La, respectively^10,22^.

The clusters that behave more like a liquid, called GUMs, are necessary to fill spaces and connect the backbone structures in MGs^10^. A lack of GUMs may result in disconnected backbone structures, decreasing the MG’s GFA. In contrast, if the concentration of GUMs is too high, the MG’s GFA may decrease because the liquid-like behavior of the GUMs would decrease the MG’s overall viscosity. Therefore, MG formation is expected to occur at preferred atomic ratios between the solute and solvent elements, with appropriate range of concentrations of GUMs. The Gaussian distribution of the atomic ratios in the Ma *et al*.’s study was consistent with the results of Inoue *et al*., who reported that AYNC_x_ had a glass structure when the Al concentration was in the range of 83–85%^19^. Therefore, the coordination number occurs over a range and is not a fixed constant. This range also overlaps with the width of the enthalpy well found in this study.

The GUMs can be assumed to form mainly within the enthalpy well, as corroborated by previous studies, in which the GUMs were found to be necessary for glass formation^10^, and where the glass was observed to form within the range of the enthalpy well^19^. The GUMs formed within the enthalpy well are expected to show liquid-like behavior based on the lower Δ*H*_vap_ within the enthalpy well. Lower Δ*H*_vap_ means the GUMs need less energy to vaporize breaking bonds with neighbor atoms on the surface, holding other conditions such as surface morphology identical. Atoms with lower Δ*H*_vap_ are expected to move more easily even in the MG’s interior because MGs have homogeneous amorphous structure. Therefore, some of the clusters formed within the enthalpy well will be the GUMs that behave more like a liquid, resulting in MGs with lower viscosity. Based on both the previous and current studies, we propose two possible explanations: (1) the concentration of GUMs that supports MG formation is specific to each composition, and (2) the GUMs form within the enthalpy well and have lower viscosity than GFMs.

The concentration of GUMs in the MG can be estimated by the weight loss shown in Fig. 2 because clusters that behave like liquids can be expected to vaporize. Although weight loss by vaporization mainly occurs from the samples surface, the GUM concentration on the surface should be identical to that of the sample interior because MGs have homogeneous amorphous structure. Thus, we can assume that the weight loss up to crystallization is proportional to the concentration of GUMs. Under this assumption, the GUM concentrations were highest at the bottoms of the enthalpy wells and decreased as the Al concentration deviated from those points. AYN was found within the enthalpy well of AYN_x_, and AYNC and AYNCL were near the edges of their enthalpy wells. Therefore, the higher GFA of AYNC and AYNCL compared to AYN was attributed to a lower concentration of GUMs, which would lead to higher viscosities for these MGs.

In addition to the GUM concentration, the GUM viscosity could also be expected to affect the composition’s GFA. Because GUMs were found to form within compositions corresponding to the enthalpy well, the lower Δ*H*_vap_ of these compositions may have led to liquid-like behavior, i.e., lower viscosity of the GUMs. The relationship between the viscosity of the GUMs and Δ*H*_vap_ can be verified by the MG viscosity, which should be proportional to that of the GUMs. The relative viscosities of the MGs can be obtained by comparing their GFAs and the driving forces required for crystallization. The viscosity and the driving force for crystallization are the main factors governing the GFA of MGs^24,25^. The driving force for crystallization (Δ*G*_α_) is proportional to the Gibbs free energy difference between the liquid and solid phases, Δ*G*_α_ = *G*_liquid_ − *G*_solid_^26^. Δ*G*_α_ is generally estimated by ${(1-{T}_{rg})}^{-2}$, in which *T*_rg_ is the reduced glass transition temperature, i.e., *T*_g_ divided by the liquidus temperature. Δ*G*_α_ of Al_84.5_Y_10_N_5.5_, Al_85_Y_8_N_5_Co_2_, and Al_86_Ni_6_Y_4.5_Co_2_La_1.5_, the compositions of which are close to AYN, AYNC, and AYNCL, were 2.68, 3.08, and 2.99, respectively (see Supplementary Table S1). The smaller Δ*G*_α_ of Al_84.5_Y_10_N_5.5_ compared to Al_85_Y_8_N_5_Co_2_ and Al_86_Ni_6_Y_4.5_Co_2_La_1.5_ means that Al_84.5_Y_10_N_5.5_ requires a lower driving force for crystallization (Δ*G*_α_). However, Al_84.5_Y_10_N_5.5_ is more easily crystallized than the others, thereby exhibiting a lesser GFA. It should be because of lower viscosity. The lower viscosity of Al_84.5_Y_10_N_5.5_ can be explained by its lower Δ*H*_vap_ because the composition occurs within the enthalpy well. The higher viscosities of Al_85_Y_8_N_5_Co_2_ and Al_86_Ni_6_Y_4.5_Co_2_La_1.5_ compared to Al_84.5_Y_10_N_5.5_ were attributed to their higher Δ*H*_vap_, which was considered to originate from their position with their respective enthalpy wells. The higher viscosities of AYNC and AYNCL may be attributed to the higher viscosity of their GUMs, which would be consistent with the result we expected from the relationship between the viscosity of the GUMs and Δ*H*_vap_. In summary, the lower concentration of the GUMs in AYNC and AYNCL was verified by the total weight loss up to crystallization and the higher viscosities was verified by the Δ*H*_vap_ values of those samples compared to the ones of AYN. Therefore, lower concentration of the GUMs with higher viscosities finally resulted in AYNC and AYNCL with higher GFA compared to AYN.

The enthalpy well has also been also substantiated by the critical volume strain, *ε*_crit_, in previously studied MGs^27,28^. The left and right edges of the enthalpy well that define its width can be explained by *ε*_crit_. When the concentration of solute elements is below the solid solution limit, the alloy system is stable. Adding solute atoms over the solid solution limit, the volume strain throughout the crystal reaches *ε*_crit_, and the alloy then destabilizes and freezes into a glass by rapid cooling^27,28^. Because adding more solute atoms is equivalent to decreasing the Al concentration in the case of Al-based MGs, *ε*_crit_ can be represented by the right edge of the enthalpy well. Once the Al concentration is higher than that at the right edge, the strain decreases below *ε*_crit_, resulting in solid-like behavior as shown in AYN_x_ in Fig. 1. At the left edge of the enthalpy well, the alloy system transforms to another stable crystalline structure such as an intermetallic compound rather than the volume strain continues to increase. Thus, the substitution of Y in AYN with Co and La increased the *ε*_crit_ of AYNC and AYNCL. The addition of 0.5 or 1.5 at.% more Al in AYNC and AYNCL compared to AYN did not reduce the total strain below *ε*_crit_. Thus, the substitution of Y in AYN with Co and/or La exerted positive and negative effects simultaneously on the achievement of a higher GFA in AYNC and AYNCL compared to AYN. The positive effect was the increased *ε*_crit_, which extended the right edge of the enthalpy well and allowed the formation of clusters that behaved like a liquid despite the high Δ*H*_vap_. In these cases, the higher Δ*H*_vap_ led to the MGs with higher viscosity and finally to higher GFA. In contrast, AYN was expected to offer a narrower range for the formation of the GUMs with higher Δ*H*_vap_, although that range was not detected in this study. In addition, within the range of proper concentration for MG formation, the lower concentration of the GUMs, also contributed to increase the viscosity of the MGs. The negative effect of the substitution on the GFA was the resulting increase in the driving force required for crystallization. However, the higher driving force was counteracted by the increased *ε*_crit_, Δ*H*_vap_, and viscosity.

## Conclusion

The enthalpy well shown in the plots of Δ*H*_vap_ against Al concentration provided insight into the GFA of Al-based MGs. The AYN sample, which was the composition with the maximum GFA among AYN_x_, was located within the enthalpy well of AYN_x_. When the Al concentration was higher than 85%, this type behaved like solid. In contrast, AYNC and AYNCL occurred near the edges of their enthalpy wells. The compositions at the right edges of their enthalpy wells behaved like liquids in spite of their higher Δ*H*_vap_. The difference in the relative positions of AYN, AYNC, and AYNCL on their enthalpy wells led to differences in Δ*H*_vap_, which was lowest in AYN, followed by AYNC, and finally AYNCL. The Δ*H*_vap_ was proportionally related to the viscosity of the GUMs in the MGs, and the GUM viscosity was considered to determine the viscosity of the MGs. Measurements of the weight loss up to crystallization confirmed the inverse proportionality of the concentration of GUMs with the enthalpy well curve. Therefore, the relatively higher GFAs in the Al-based MGs was attributed to higher viscosities that resulted from the higher viscosity and lower concentration of the GUMs in these materials.

## Methods

### MG ribbons synthesis

Al-based MG ribbons were fabricated by rapid solidification with compositions of AYN_*x*_, AYNC_*x*_, and AYNCL_*x*_. The master alloys were prepared by arc-melting high-purity elements (purity > 99.9%; RND Korea). Using these master alloys, MG ribbons were produced by melt-spinning under Ar gas in a vacuum chamber (base pressure: 10^−5^ Torr). The rotation speed of a copper wheel (24.5-cm diameter) was 3000 revolutions per minute. The crystallinities and thermal properties of the amorphous ribbons were confirmed using differential scanning calorimetry (Q2000, TA Instruments) and X-ray diffraction (Bruker D8 ADVANCE, Bruker), as shown in Supplementary Figs. S2 and S3 and Tables S2–S4. The ribbon composition was confirmed using an energy dispersive X-ray spectroscope connected to a scanning electron microscope (FEI Helios 600i).

### Vapor pressure measurement

The vapor pressures of the MG ribbons were measured via isothermal analysis according to Eqs. (1) and (2). The weight losses of the samples were determined using thermogravimetric analysis (TA Instruments Q500) at eight constant temperatures under *T*_g_: 478, 488, 498, 508, 518, 528, 538, and 548 K. In the beginning of the isothermal analysis, the temperature of the thermogravimetric analysis chamber was maintained at 300 K for 5 min and then increased to the target temperature using the Equilibrate method. The Equilibrate method helps to increase temperature to a target temperature as quickly as possible minimizing overshooting in the TGA machine. Finally, temperature sustained at the target temperature for 15 min to calculate dm/dt in Eq. (2). The weight loss was measured three times using three ribbons at each target temperature. The sample weight and area for each scan were approximately 3 mg and 30 mm^2^, respectively. Images of the MG ribbon segments on the thermogravimetric analysis pan were obtained, and the area of the top ribbon surface exposed to air (*Z*) was measured using Photoshop (Adobe CS6, Adobe Systems). The measurement error caused by the thermogravimetric analysis equipment was verified by measuring the weight change of an empty platinum pan using the same heating profile. Although the equipment’s sensitivity as reported by the manufacturer is 0.1 μg, the measurement error was approximately 1.5 ± 1.4 μg.

The effective weight loss was used to estimate Δ*H*_vap_ of the Al-based MGs. The effective weight loss *dm*_eff_ is the measured weight loss subtracted by the average value of the measurement error as shown in Eq. (3).

3 $$d{m}_{eff}=d{m}_{MG}-1.5(unit:\mu g)$$

Using *dm*_eff_ not only minimizes the effect of the equipment error but also enables determination of the relative Δ*H*_vap_ of Al-based MGs with respect to ΔH_sub_ of c-Al. We therefore assumed 1.5 μg of weight loss of c-Al by sublimation. For the measurement, a reference value was required because the Δ*H*_vap_ of the MGs had not been estimated. We chose c-Al as the reference based on the fact that approximately 85% of the MGs under investigation was Al, and the theoretical value of Al (Δ*H*_*sub*_) was previously reported as in Eq. (4)^29^. Thus, the Δ*H*_vap_ of the MG calculated using the effective weight loss was the relative value with respect to the ΔH_sub_ of c-Al.

4 $$\log ({\rm{P}})=14.465-\frac{17342}{{\rm{T}}}-0.7927\times \,\log ({\rm{T}})$$

To validate this assumption, proper values of α in Eq. (2) were necessary to compensate for the small amount of weight loss caused by the sublimation of c-Al, and Supplementary Table S5 lists the proper values of α as determined using Eq. (1) and Eq. (2). From these equations, ΔH_sub_ of c-Al was found to be 320 kJ/mol. When α = 1 in Eq. (2), the estimated weight loss of a c-Al sample with an area of 30 mm^2^ (typical of the samples analyzed here) was approximately 4.8 × 10^−19^ μg after annealing at 478 K for 15 min. The weight loss was too low to be detected in the thermogravimetric analysis. This calculation matched the weight loss of the c-Al foil with the area of about 30 mm^2^, within the error range of the equipment, in the actual thermogravimetric measurement of the c-Al. To achieve the measurable weight loss, 1.5 μg, the sample area of the c-Al foil should be 3 × 10^18^ times larger when the isothermal annealing is conducted at 478 K. Because such a large sample cannot be loaded in the thermogravimetric analysis equipment, the factor of the sample area of c-Al was reflected using α, the values of which at different temperatures are summarized in Supplementary Table S5.

The weight loss of each MG sample was measured three times at each temperature. Using the α and *dm*_eff_, the average and standard deviation of *P*_*vap*_ at the temperatures were obtained from Eq. 4, and Δ*H*_vap_ of the MG sample was finally estimated by linear fitting with Origin software. We used the Instrumental function in the Origin software to minimize the influence of the machine error, whereby *P*_*vap*_ values with smaller standard deviations could be weighted more heavily into the linear fitting. Supplementary Figs. S4–S6 show the linearly fitted *P*_*vap*_ vs. 1/*T*. The strategy of determining the relative values of Δ*H*_vap_ with respect to Δ*H*_*sub*_ of c-Al was thus found to produce more realistic values for the Δ*H*_vap_ of the MGs compared to those previously reported^30^.

### Hardness and elastic modulus measurements

The hardness and elastic moduli of the MG ribbons were measured using a nanoindenter (Zwick Roell, ZHN nanoindenter, Germany) with a pyramidal Berkovich tip. Three samples were prepared from each composition. Natural rosin light (D’Addario, VR200, USA) was used to attach the samples on the holder. The measurements proceeded following ISO 14577^31^. This method consists of the following steps: loading, creep, unloading, holding, and final unloading. The maximum force for loading was set to 100 mN for 20 s, and then the creep force was set to 100 mN for 15 s, with unloading at 10 mN for 10 s, holding at 10 mN for 15 s, and final unloading at 0.06 mN for 3 s. The Oliver and Pharr method was applied to calculate the hardness and reduced elastic modulus^32^. We set the Poisson’s ratio of the MG ribbons at 0.33, which is that of pure aluminum. Supplementary Fig. S7 shows a typical load-unload nanoindentation curve.

## Supplementary information

Supplementary Information.
