# Supplementary material for: Investigation of glass forming ability of Al-based metallic glasses by measuring vaporization enthalpy
Source: Sci Rep. 2020 Mar 5;10:4162. doi: 10.1038/s41598-020-61134-8 (PMC7058064; doi:10.1038/s41598-020-61134-8)
Supplement: Supplementary file 1 — Supplementary Information. [file 41598_2020_61134_MOESM1_ESM.docx]

Supplementary Information

**Investigation of glass forming ability of Al-based metallic glasses by measuring vaporization enthalpy**

Byeong-uk Min, Jun-ho Lee, Ho-jun Park, Gyu-tae Jeon, Jae Im Jeong, Sung Hyuk Lee, and Suk Jun Kim^*^

*School of Energy, Materials and Chemical Engineering, Korea University of Technology and Education, Cheonan, 31253, Korea*

**Corresponding author, e-mail:* [skim@koreatech.ac.kr](mailto:skim@koreatech.ac.kr)

Tables

Table S1. Estimated ΔG_α_ of Al_84.5_Y_10_N_5.5_, Al_85_Y_8_N_5_Co_2_, and Al_86_Ni_6_Y_4.5_Co_2_La_1.5_ calculated using $\left( 1-T_{rg} \right)^{-2}$. The *T*_rg_ was obtained using previously reported *T*_g_ and *T*_l_.

| Sample | *T*_g_ (K) | *T*_x_ (K) | *T*_l_ (K) | *T*_rg_ (K) | Δ*G*_α_ |
| --- | --- | --- | --- | --- | --- |
| Al_84.5_Y_10_N_5.5_ ^15^ | 480 | 517 | 1232 | 0.39 | 2.68 |
| Al_85_Y_8_N_5_Co_2_ ^24^ | 530 | 554 | 1231 | 0.43 | 3.08 |
| Al_86_Ni_6_Y_4.5_Co_2_La_1.5_ ^13^ | 505 | 513 | 1197 | 0.42 | 2.99 |

Table S2. *T*_g_ and *T*_x_ of AYN*_x_*.

| ***Al concentration (at.%)*** | ***T_g_* (K)** | ***T_x_* (K)** |
| --- | --- | --- |
| *82.6* | *536* | *556* |
| *83.2* | *545* | *562* |
| *83.8* | *539* | *553* |
| *84.3* | *538* | *556* |
| *84.7* | *538* | *557* |
| *85.2* | *None* | *None* |
| *85.4* | *None* | *None* |

Table S3. *T*_g_ and *T*_x_ of AYNC*_x_*.

| ***Al concentration (at.%)*** | ***T_g_* (K)** | ***T_x_* (K)** |
| --- | --- | --- |
| *82.9* | *541* | *565* |
| *83.3* | *534* | *561* |
| *83.8* | *517* | *539* |
| *84.7* | *499* | *517* |
| *85.0* | *534* | *557* |
| *85.2* | *513* | *528* |
| *85.4* | *533* | *556* |

Table S4. *T*_g_ and *T*_x_ of AYNCL*_x_*.

| ***Al concentration (at.%)*** | ***T_g_* (K)** | ***T_x_* (K)** |
| --- | --- | --- |
| *82.4* | *None* | *None* |
| *83.5* | *530* | *550* |
| *84.1* | *530* | *552* |
| *85.3* | *485* | *504* |
| *85.8* | *489* | *509* |
| *86.2* | *500* | *516* |
| *87.1* | *None* | *None* |

Table S5. Theoretical vapor pressure and corresponding weight loss of c-Al with a surface area of 30 mm^2^ during isothermal analysis for 15 min at various temperatures, and α values required to obtain the theoretical vapor pressure that induces weight loss of 1.5 μg.

| **Temp.(K)** | **478** | **488** | **498** | **508** | **518** | **528** | **538** | **548** |
| --- | --- | --- | --- | --- | --- | --- | --- | --- |
| **Vapor pressure (Pa)** | 1.1e–24 | 6.3e–24 | 3.2e–23 | 1.5e–22 | 6.8e–22 | 2.9e–21 | 1.2e–20 | 4.5e–20 |
| **Weight loss (μg)** | 4.8e–19 | 2.6e–18 | 1.3e–17 | 6.1e–17 | 2.7e–16 | 1.1e–15 | 4.6e–15 | 1.7e–14 |
| **α** | 3.14e+18 | 5.82e+17 | 1.15e+17 | 2.44e+16 | 5.49e+15 | 1.31e+15 | 3.29e+14 | 8.69e+13 |

Figures


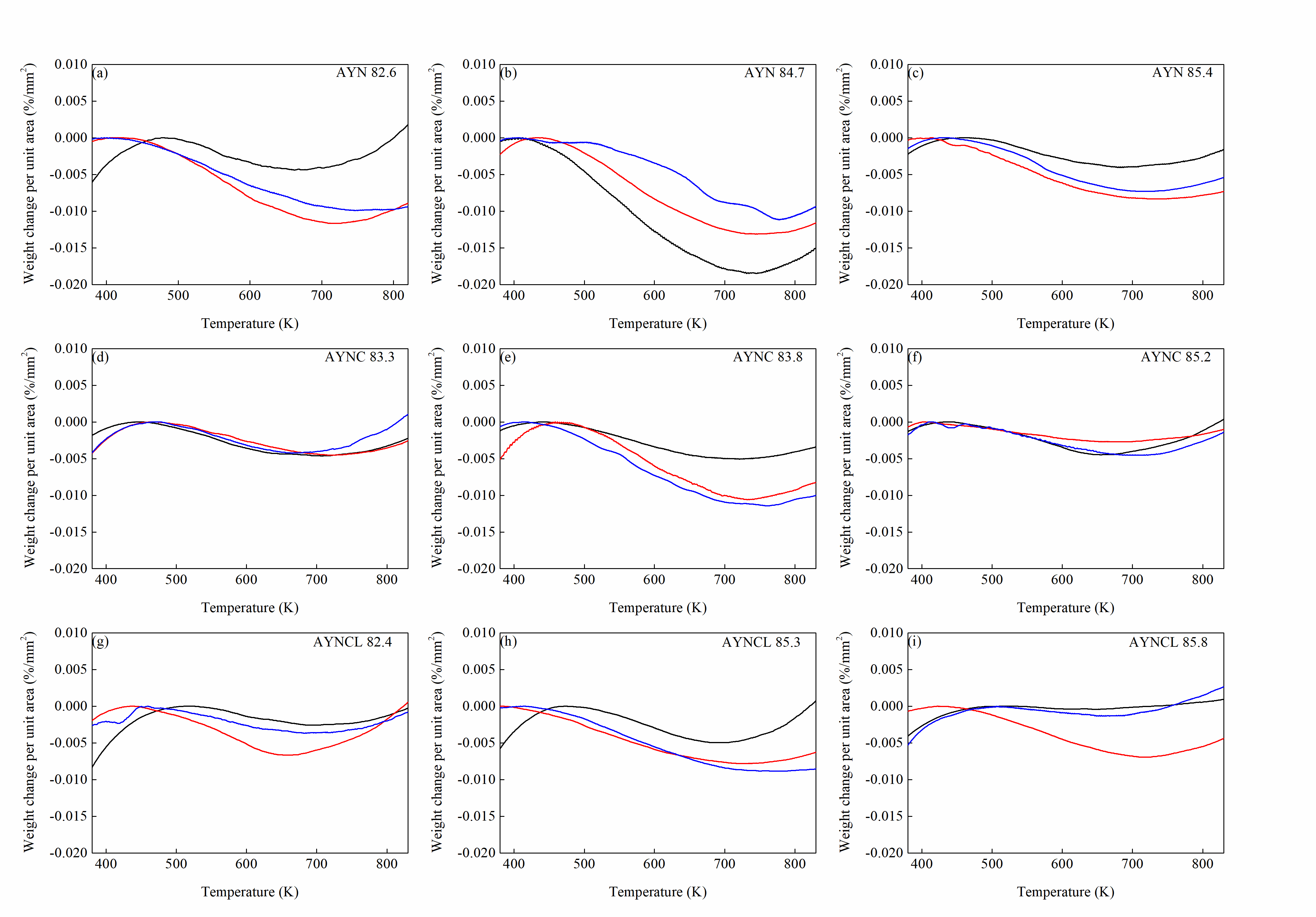


Figure S1. Weight loss of the MGs per sample area measured under continuous heating. Samples with compositions at the left edge, bottom, and right edge of the enthalpy wells of (a,b,c) AYN*_x_*, (d,e,f) AYNC*_x_*, and (g,h,i) AYNCL*_x_*.


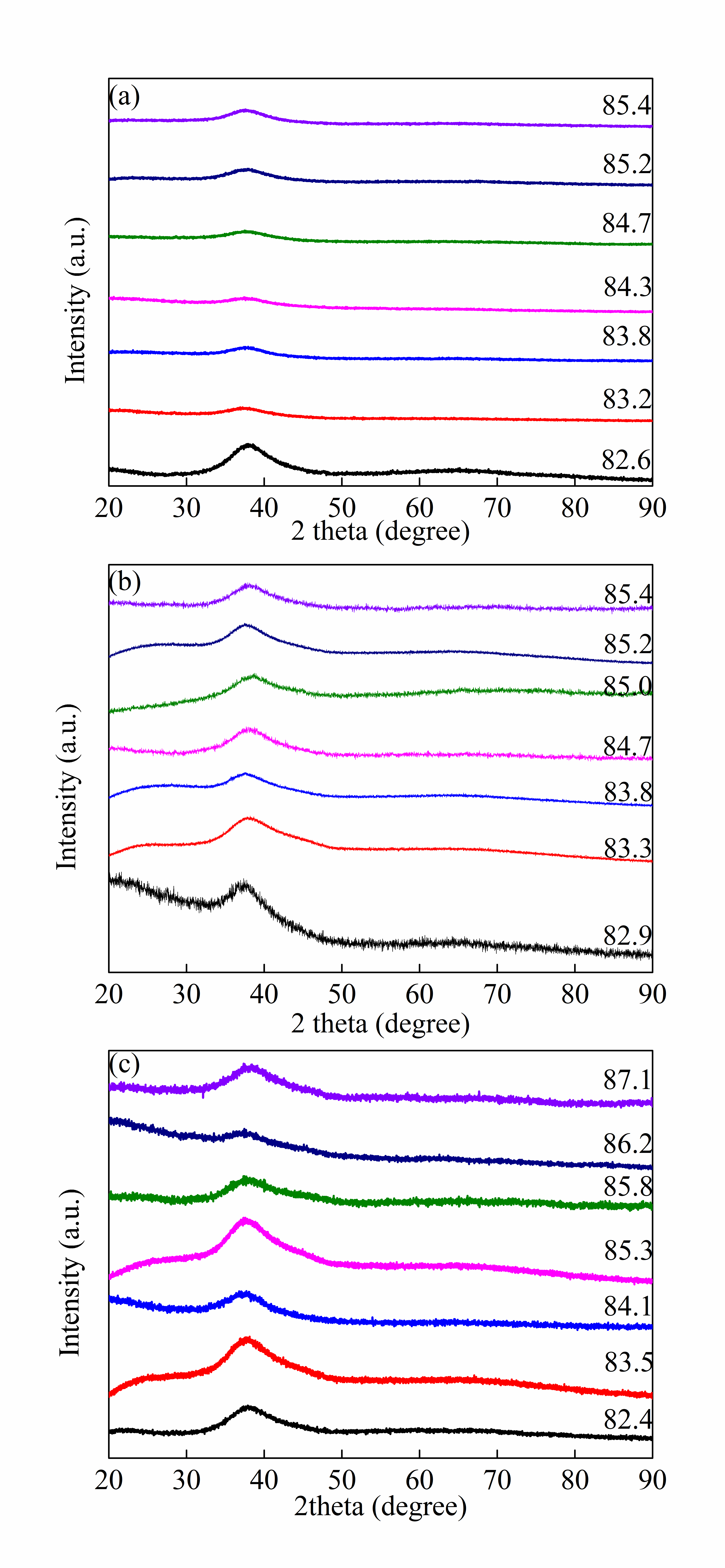


Figure S2. X-ray diffraction profiles of AYN*_x_*, AYNC*_x_*, and AYNCL*_x_* with various *x*.


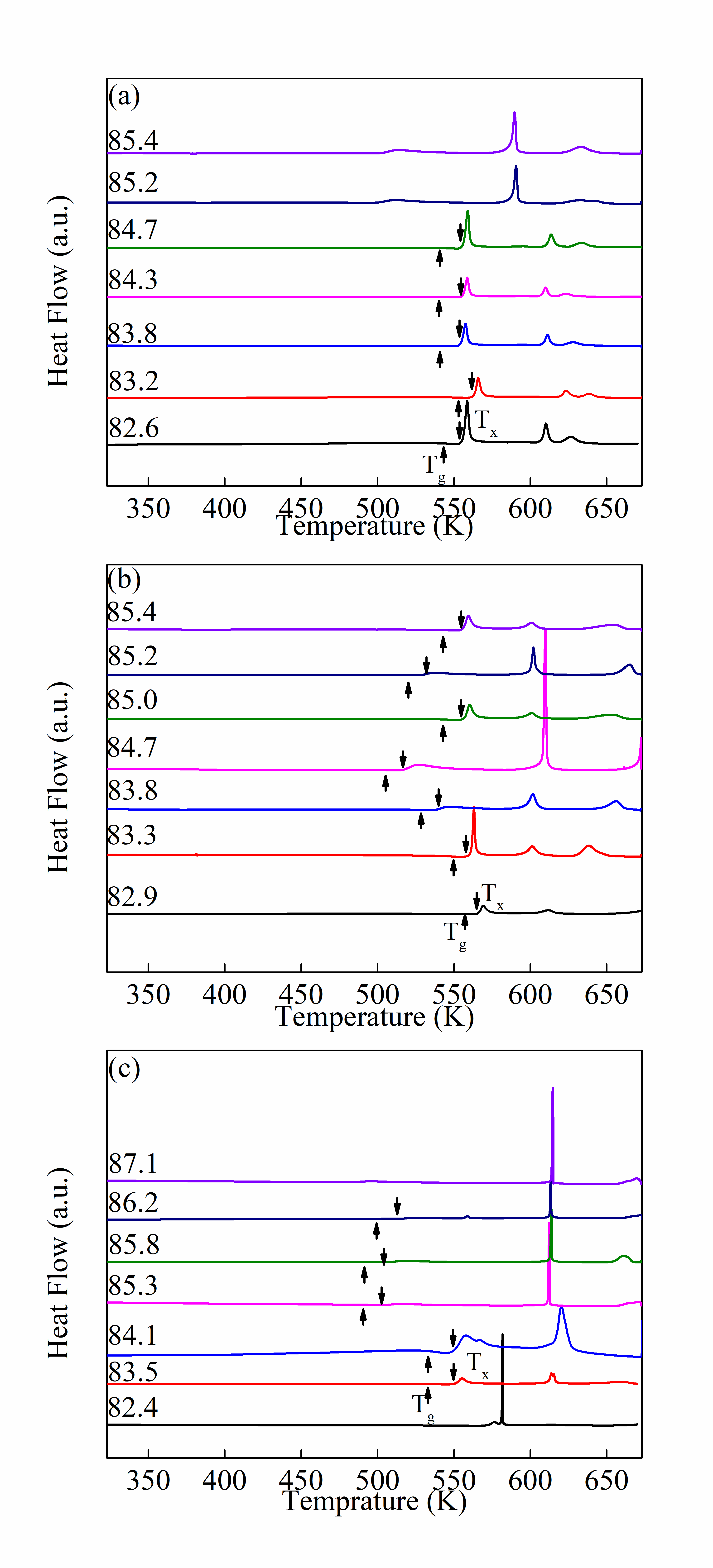


Figure S3. Differential scanning calorimetry profiles of AYN*_x_*, AYNC*_x_*, and AYNCL*_x_* with various *x*.

Figure S4. Linear fitting of *P_vap_* vs. 1/*T* of AYN*_x_*.


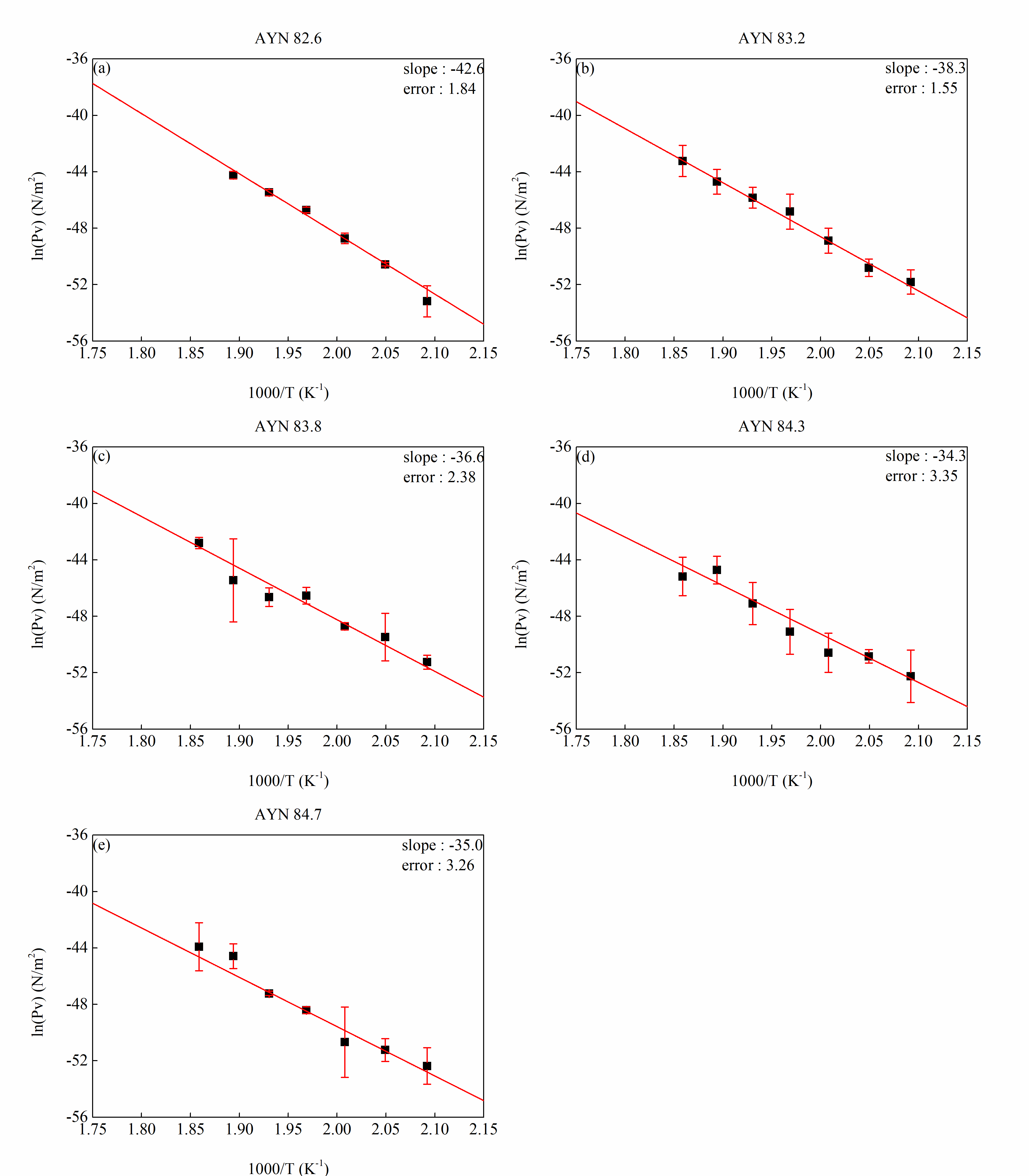

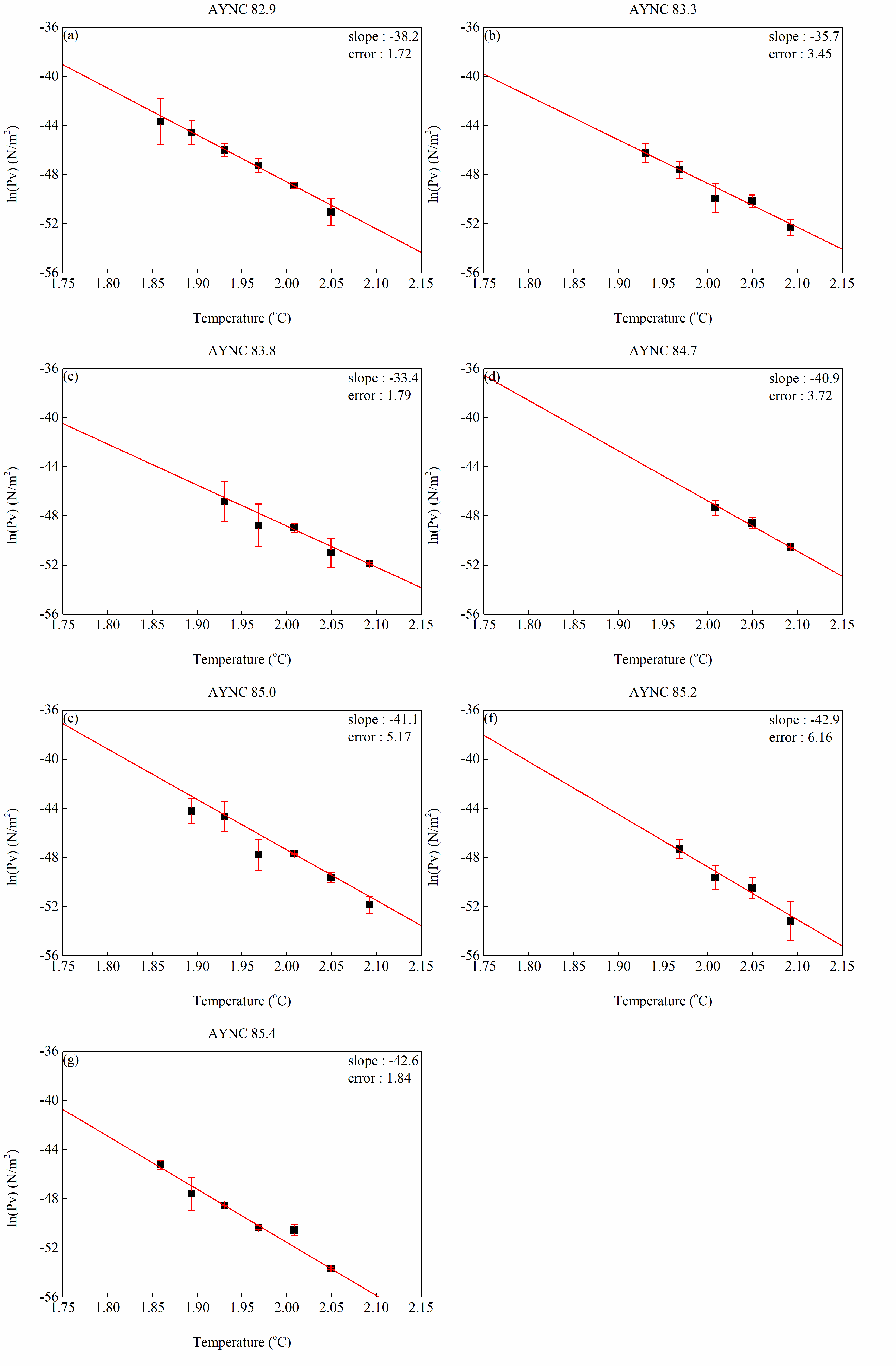


Figure S5. Linear fitting of *P_vap_* vs. 1/*T* of AYNC*_x_*.


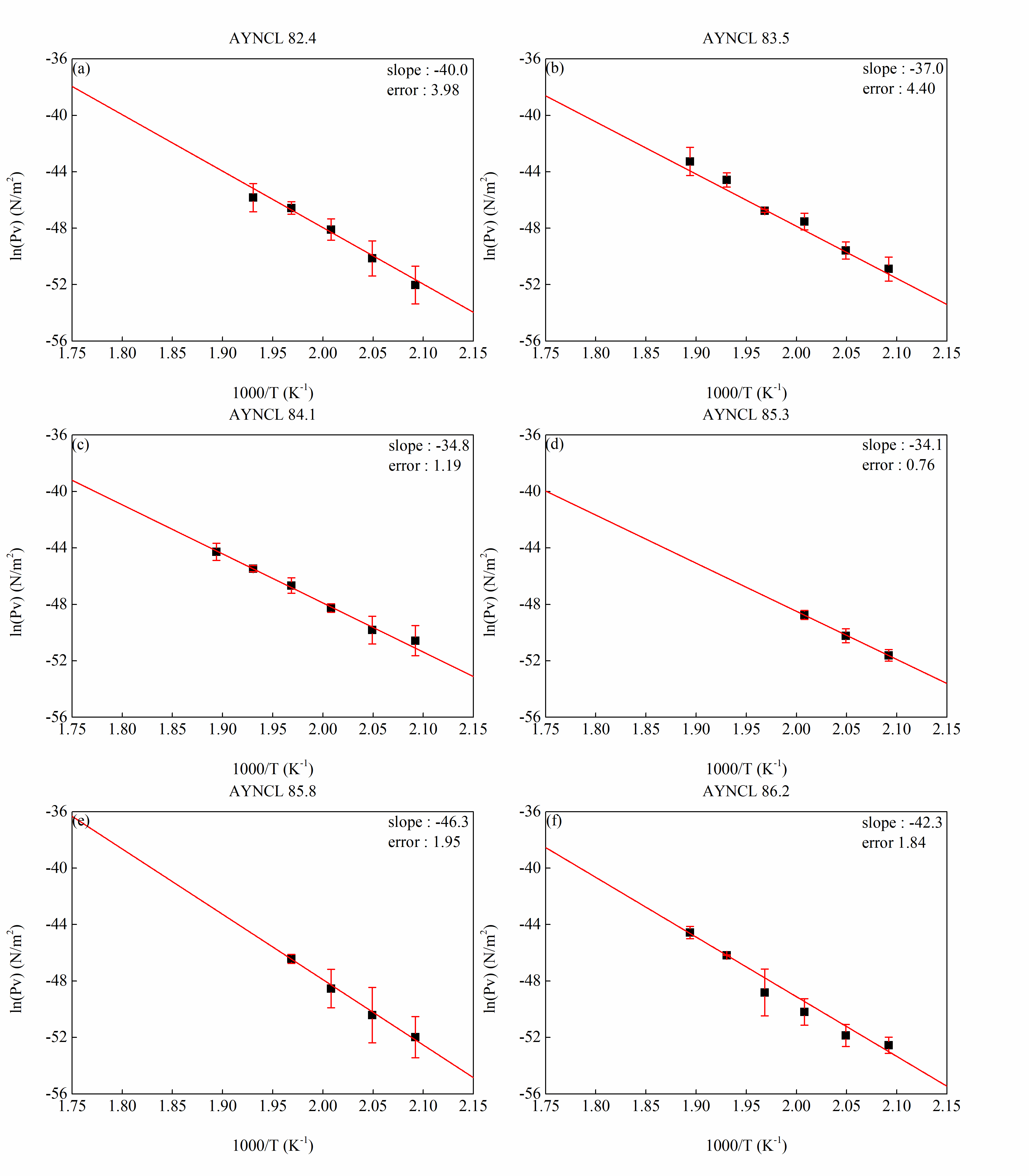


Figure S6. Linear fitting of *P_vap_* vs. 1/*T* of AYNCL*_x_*.


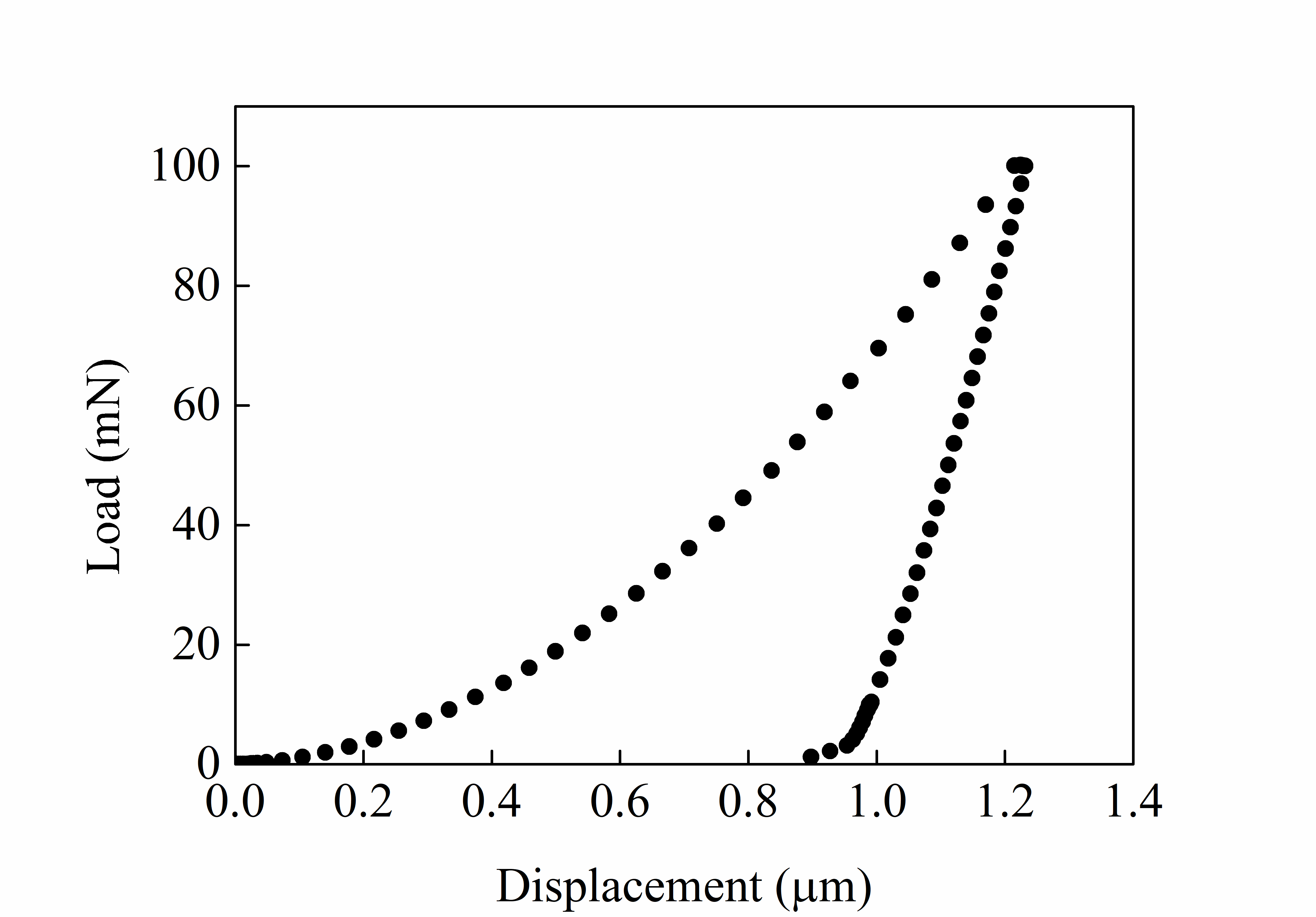


Figure S7. Load-unload nanoindentation curve of AYNC_-0.3_.
